# Biodegradation of triclosan under non-sterile conditions by co-culture of *Bacillus licheniformis* and *Lysinibacillus fusiformis*

**DOI:** 10.1007/s00449-026-03331-9

**Published:** 2026-04-18

**Authors:** Elanur Dasdemir, Mesut Taskin, Hakan Ozkan

**Affiliations:** Department of Molecular Biology and Genetics, Science Faculty, Ataturk University, Erzurum, Turkey

**Keywords:** Triclosan, Biodegradation pathway, Co-culture, Toxicity, Non-sterile process

## Abstract

Microorganisms, especially their co-cultures, that can degrade pollutants in non-sterile wastewater without requiring external nutrient supplementation, are important for sustainable wastewater treatment practices. This study aimed to (1) perform biodegradation of triclosan (TCS) using co-culture of locally isolated *Bacillus licheniformis* AEM2 (GenBank: PQ856279) and *Lysinibacillus fusiformis* AEM5 (GenBank: PQ856280), (2) analyze degradation byproducts and biodegradation pathway of TCS, (3) examine in vitro cytotoxicity of degradation byproducts, and (4) develop a non-sterile culture process for TCS biodegradation. In mono-culture strategy, TCS-containing mineral salt broth medium (MSBM) was inoculated with 1 mL of preculture of either AEM2 or AEM5. In co-culture strategy, two bacteria were simultaneously inoculated into the culture media at the different inoculum volumes (between 0.25, 0.50, and 0.75 mL/100 mL for each bacterium, with a total inoculum volume of 1 mL). The co-culture C (0.25 mL of AEM2 pre-culture + 0.75 mL of AEM5 pre-culture) (80.62% degradation efficiency) exhibited higher TCS degradation potential than mono-cultures and other co-culture formulations (*p* < 0.05). Under the optimized culture conditions (initial pH 7.0, temperature 25 °C and incubation time 72 h), the co-culture C achieved complete degradation (100%) of 10 mg/L TCS in MSBM. The main degradation products were low-chlorinated or chlorine-free phenolic intermediates. Oxygenases and dehalogenases were predicted to be responsible for biodegradation of TCS. In contrast to the parent compound TCS, its degradation products did not cause a statistically significant cell toxicity within the concentration range of 5–80 µg/mL (*p* > 0.05). The co-culture C could degrade 88.55% of 10 mg/L TCS within 72 h in non-sterile wastewater, which no external nutrients were added. To the best of our knowledge, this study investigates for the first time the TCS biodegradation potential of *B. licheniformis* and *L. fusiformis*, and evaluates the effectiveness of a non-sterile, bacteria-based co-culture system for TCS remediation.

**Supplementary Information:**

The online version contains supplementary material available at 10.1007/s00449-026-03331-9.

## Introduction

Personal care products (PPCPs), which are indispensable for humans, include soaps, creams, toothpastes, deodorants and disinfectants. After these products are discarded of after their use, they pass into sewerage systems and are treated in wastewater treatment plants. However, it has been reported that the actual removal processes in wastewater treatment plants are limited [1–4].

Triclosan (TCS), also known as 2,4,4‘-trichloro-2’-dihydroxy diphenyl ether, is one of the important PPCPs. Due to its antimicrobial properties, TCS is widely used as an ingredient of pharmaceutical and personal care products (mostly antibacterial soaps, disinfectants, toothpaste and some cosmetics) [5, 6].

TCS has been reported to cause contact dermatitis, some allergic reactions, and low-level endocrine disrupting effects in humans [7, 8]. Animal studies have shown that TCS can increase fatty liver disease and lead to developmental disorders [9, 10]. In addition, this compound has been reported to cause bioaccumulation and endocrine disrupting effects in fish, as well as oxidative stress, DNA damage, and metabolic disorders in algae and benthic organisms [11, 12]. Furthermore, it has been noted that this compound contributes to the development of antibiotic resistance and can transform into more toxic compounds, such as dioxin-like products, when it reacts with sunlight and water [8, 13, 14]. For these reasons, TCS is considered a chemical that requires careful evaluation [7, 8, 13].

TCS is commonly detected in surface waters, wastewater, and sediments, its removal from these environmens is considered a critical necessity. Accordingly, advanced oxidation, adsorption, and biodegradation methods are used to remove TCS from environmental sources [1, 15–18]. In biodegradation of TCS, fungi and bacteria can be used. To date, some bacterial strains such as *Citrobacter freundii* KS2003, *Pseudomonas aeruginosa* KS2002, *Enterobacter cloacae*, *Bacillus* sp. DL4 and *Dyella* sp. have been reported to capable of degrading TCS [19–23]. However, it is possible to say that discovering new bacteria that can degrade TCS with higher efficiency and do not create toxic degradation byproducts may offer advantages in the removal of TCS from wastewater environments. For instance, TCS-degrading potentials of *Bacillus licheniformis* and *Lysinibacillus fusiformis* strains have not been studied yet.

Biodegradation of toxical compounds is performed by using monoculture (only one microorganism species) or co-culture (mixed culture of two or more microorganism species); however, it is known that co-cultures generally provide higher biodegradation efficiency when compared to monoculture [24, 25]. So far, the biodegradation of various toxic substances has been achieved by using co-cultures [26–31]. However, co-culture technique for bacterial biodegradation of TCS has not been tested in the literature.

Microorganisms can be cultivated in sterile or non-sterile cultures for the biodegradation of toxic pollutants or the production of valuable metabolites [32–37]. In a non-sterile culture technique, the target microorganism is directly inoculated into the non-sterile culture medium or non-sterile wastewater environment. After the inoculation, the inoculated microorganism is anticipated to become more prevailing over endogenous microorganisms of non-sterile medium or external contamination during cultivation period. In the non-sterile culture technique, some simple methods are harnessed to make the target microorganism dominant, such as keeping the inoculation volume of the target microorganism high and adjusting the pH of the medium according to the target microorganism. In a non-sterile culture process, the culture medium or wastewater medium is not sterilized and the control of external contamination is not required [32, 33, 38, 39]. This process makes possible energy saving in a wastewater treatment system or bioreactors with large volume. Up to now, diverse microorganisms have been evaluated for their potency to degrade different toxic organic pollutants in non-sterile biological treatment process [37, 40–44]. Similarly, existing studies on microbial degradation of TCS have been conducted exclusively under sterile laboratory conditions, which do not adequately reflect real wastewater environments. Conversely, the biodegradation of TCS by bacteria in a non-sterile culture system, where competition with indigenous microorganisms occurs, has not been evaluated.

In short, there are some gaps in the literature regarding the microbial biodegradation of TCS: The lack of studies on *B. licheniformis* and *L. fusiformis*, the absence of co-culture and non-sterile culture strategies, and the unassessed toxicity of degradation products. Therefore, the present study aims to address these gaps by (1) investigating the TCS-degrading potential of mono-and co-cultures of locally isolated *B. licheniformis* and *L. fusiformis* (2) identifying the degradation products and proposing a possible biodegradation pathway, (3) assessing the in vitro cytotoxicity of the resulting degradation products, and (4) developing a non-sterile culture process without requiring external nutrient supplementation with potential applicability to real wastewater treatment systems.

## Materials and methods

### Isolation of triclosan-degrading bacteria

Bacteria capable of degrading triclosan (TCS) were isolated from the activated sludge of a wastewater treatment system in Erzurum, Turkey. Precautions were taken during sludge sample collection to encumber unwanted-microbial contamination from external environment. Hence, a sterile wide-mouthed glass bottle was submerged in the activated sludge, its cap was opened within the sludge, and after collecting the sample, the cap was closed again while still in the sludge. After being transported to our laboratory in a cooling box, one gram sample from activated-sludge was added into another sterile bottle containing 100 mL of sterile- saline water, and the bottle was shaken vigorously for 30 s. After the suspension was serially diluted, 0.5 mL of the prepared dilution samples were spreaded on the petri dishes containing MSAM (mineral salt agar medium) (Omeroglu et al., 2025). This medium (pH 8.0) included KH_2_PO_4_ (1 g/L), (NH_4_)SO_4_ (1.5 g/L), MgSO_4_ (0.2 g/L), NaCl (0.5 g/L), CaCl_2_ 2H_2_O (0.02 g/L), NaHCO_3_ (0.03 g/L)], ZnSO_4_ 7H_2_O (0.02 g/L), MnCl_2_ 4H_2_O (0.1 g/L), FeSO_4_ (0.005 mg/L), CuSO_4_ 5H_2_O (0.3 g/L), agar (20 g/L) and TCS (50 mg/L). In this medium, TCS was used as sole carbon source in the medium. Petri-dishes were incubated aerobically at 25 °C for 24 h. After the incubation period was completed, developed-bacterial colonies on petri dishes were subcultured and purified. The isolates were coded and then employed for the next stages of the study.

### Screening of triclosan-degrading bacteria

In order to evaluate the potential of the isolates to degrade TCS, their pre-cultures were firstly prepared. For this purpose, the isolates were grown aerobically in Luria-Bertani (LB) broth medium at 25 °C for 24 h. Afterwards, 1 mL of pre-cultures (OD_600_=1.0) were inoculated into 250 mL flask containing 100 mL of mineral salt broth medium (MSBM) supplemented with 10 mg/L TCS. The flasks were placed in a shaking incubator and incubated at 25 °C for 24 h. After the incubation, the amount of TCS in the cultures were determined spectrophotometrically according to the previously described method [45]. In brief, the culture was centrifuged (3800 x *g* for 10 min) using a centrifuge (Beckman Coulter Allegra X-30R), and the supernatant was diluted with distilled water to obtain absorbance values ​​that corresponded to the linear range of the calibration curve (It was prepared using known concentrations of TCS). The absorbance of samples was measured at 475 nm, and TCS concentration was calculated by comparing the absorbance values of the sample to the standard curve.

The isolates with high TCS degradation capacity were selected based on the result of spectrophotometric measurement; however, their TCS degradation capacity were also analysed using a reverse-phase HPLC system (Shimadzu LC-20AD). For this purpose, the culture supernatants were subjected to ethyl acetate-extraction (1:1, v/v) at 150 rpm for 6 h. The organic phase obtained after extraction was dewatered with Na_2_SO_4_ to remove residual water. In the last step, the organic phase was evaporated to dryness by evaporator. The concentrated TCS residues were dissolved in 1 mL methanol. The prepared sample was analysed by a reverse-phase HPLC system (Shimadzu LC-20AD) equipped with a photodiode array (PDA) detector (SPD-M20A). Separation was achieved on a Kinetex^®^ C18 column (250 × 4.6 mm i.d., 5 μm particle size, 100 Å pore size; Phenomenex, USA). The mobile phase consisted of solvent A and solvent B mixed in an isocratic ratio of 15:85 (v/v). The flow rate was set at 0.8 mL/min. The column temperature was maintained at 25 °C throughout the analysis. The total run time was 15 min. Detection was carried out at 250 nm with a bandwidth of 4 nm. The PDA detector operated with a reference wavelength of 350 nm, and the cell temperature was maintained at 40 °C.

The percentage of TCS degradation was calculated according to the following Eq. (1).

1 $$\:Degradation\:ratio\:\left(\%\right)=\left(\frac{\mathrm{C}0-\mathrm{C}1}{\mathrm{C}0}\right)\times \:100$$

Where C0 is the initial TCS concentration (mg/L) and C1 is the final TCS concentration (mg/L).

### Molecular identification of TCS-degrading bacteria

Two isolates exhibiting higher potential to degrade TCS were molecularly identified based on 16 S rRNA sequence analysis. For this purpose, firstly, genomic The extraction of DNA from bacterial cells was performed using Promega Wizard^®^ Genomic DNA Purification Kit (A2360) protocol. For the amplification of 16 S rRNA gene, the following primers were used: 27 F (5′-AGAGTTTGATCCTGGCTCAG-3′) and 1492 R (5′-GGTTACCTTGTTACGACTT-3′). The PCR product was cloned into *Escherichia coli* strain JM101 using pGEM-T Easy Cloning Vector (Promega, Southampton, UK) according to the manufacturer’s instructions. After cloning, samples were sequenced at Macrogen Company (Amsterdam, The Netherlands). The GenBank accession number was determined by comparison with other bacterial sequences available in GenBank and EzTaxon (http://blast.ncbi.nlm.nih and http://www.eztaxon.org). A Neighbour-Joining phylogenetic tree based on the 16 S rRNA gene sequencing data was then constructed using Mega 11 software.

### Comparison of TCS biodegradation abilities of mono-and co-cultures of selected bacteria

In this step, mono-cultures and co-cultures of two bacteria were tested for TCS degradation. For this purpose, pre-cultures of bacteria were prepared in LB broth medium and the optical density of each pre-culture was setted to 1.0 absorbance at 600 nm. To prepare co-culture from two isolates, different volumes of precultures of two isolates were combined.

Mono-culture and co-cultures formulations were designed as follows.

- Mono-culture A: It was inoculated with preculture (1 mL) of isolate I.
- Mono-culture B: It was inoculated with preculture (1 mL) of isolate II.
- Co-culture C: It was inoculated with preculture (0.25 mL) of isolate I and preculture (0.75 mL) of isolate II.
- Co-culture D: It was inoculated with preculture (0.5 mL) of isolate I and preculture (0.5 mL) of isolate II.
- Co-culture E: It was inoculated with preculture (0.75 mL) of isolate I and preculture (0.25 mL) of isolate II.

The total size of pre-cultures was adjusted to 1 mL/100 mL. After 250 mL-flasks containing 100 mL of sterile MSBM supplemented with 10 mg/L TCS were inoculated with the pre-cultures, they were incubated at 25 °C for 24 h. After the incubation, the cultures were centrifuged at 3800 x *g* for 10 min and the obtained supernatants were used to determine the degradation ratio of TCS. In this stage, the amount of TCS in the supernatants was determined spectrophotometrically. Degradation efficiency was presented as percentage removal for comparison clarity.

### Effects of culture parameters on TCS biodegradation potential of co-culture

After determining the most favorable co-culture combination, the subsequent experiments were focused on evaluating the effects of medium pH (6, 7, 8, 9 and 10), temperature (15, 20, 25, 30, 35, 40, 45, 50, 55 and 60 °C), initial TCS concentration (5, 10, 20, 30, 40 and 50 mg/L) and incubation time (24, 48, 72 and 96 h) on the TCS biodegradation potential of the co-culture. In the experiments, one mL of co-culture was employed to inoculate the sterile MSBM supplemented with TCS. After the inoculation, the flasks were incubated in a shaking incubator at 150 rpm under sterile conditions. At the end of the specified incuabtion period, the amount of TCS in the culture sepernatants was determined spectrophotometrically and the degradation efficiency was presented as percentage removal for comparison clarity.

### Analysis of degradation byproducts and degradation pathway of TCS

To analyze TCS degradation products, the culture was centrifuged at 3800 x *g* for 10 min. After the pH of the culture supernatant was adjusted to 2.0 using 1 M H_2_SO_4_, it was extracted with ethyl acetate (1:1, v/v) at 150 rpm for 6 h. The organic phase was evaporated and the concentrated TCS residues were dissolved in methanol. After BSTFA (20 µL) was added, the mixture was incubated at 50 °C for 1 h. GC-MS analysis was performed to analyze the degradation metabolites in the mixture.

Predicting the biodegradation pathway of TCS was performed mainly based on the types of the chemical reactions, which catalyze the formation of TCS degradation metabolites. Moreover, the laccase and manganese peroxidase activities in the culture were also analyzed to elucidate their possible role in TCS degradation. Determination of laccase and manganese peroxidase activities was performed according to the method described in our previous study [39].

### Toxicity analysis of triclosan and its degradation products

The supernatant from the treatment culture was extracted with ethyl acetate (1:1, v/v) at 150 rpm for 6 h. The ethyl acetate fraction was dewatered with Na_2_SO_4_ and then was evaporated to dryness using a rotory evaporator. The concentrated final material (TCS residues) was weighed and quantified. For the toxicity analysis, the final material was dissolved in 1 mL DMSO (1%) and this solution was used as stock. The stock solution was then diluted with DMSO (1%) to prepare different dilution groups (5–160 µg TCS residues/mL DMSO) from TCS residues. Similarly, dilution samples were prepared from pure TCS with 1% DMSO at the same concentrations. Finally, the dilutions samples were evaluated for their potantial cytotoxic properties.

Cytotoxicity level was evaluated by water-soluble tetrazolium salt (WST-1) method measuring mitochondrial reductase activity. L929 (mouse fibroblast cell line, ATCC CCL-1) cells used in the viability assay were incubated in DMEM/F-12 medium supplemented with 10% fetal bovine serum (FBS), L-glutamine and 100 U/mL penicillin/streptomycin (at 37℃ in a 5% CO_2_ incubator). The culture medium was changed every 2 days. A concentration of 3 × 104 cells/mL was seeded in 96-well plates. The cultured cells were treated with the dilution samples (containing TCS or TCS residues) and then were re-incubated at 37 °C for 24 h. Cells not exposed to TCS or its degradation products were used as the control (C) group. Optical density was measured at 450 nm in a multi-well plate reader (Thermo Scientific™ Multiskan™ Microplate Spectrophotometer). The viability was given as percentage (%).

### Biodegradation of TCS by co-culture in non-sterile wastewater-based medium

In this step of the study, TCS-degrading capacity of the co-culture was tested sterile or non-sterile wastewater-based medium under non-sterile conditions. Experiments for TCS degradation were carried out in 250 mL flasks containing 100 mL wastewater-based media. Three different media were prepared for the experiments. All media contained only wastewater and 10 mg/L TCS and no nutrients (minerals, carbon source, nitrogen source, etc.) were added to these media. The pH of the prepared media was not adjusted (the initial pH of the wastewater was 7.12). Medium I was sterilised and the co-culture was then inoculated into this medium. Medium II was not sterilized and directly inoculated with co-culture. Medium III was not sterilized and the co-culture was not inoculated. In addition, the flasks containing non-sterile wastewater media (medium II and medium III) were not covered with cotton plug and then left to the incubation under non-sterile conditions opened to environment. At this stage of media testing, the temperature of the shaking incubator was kept constant at 25 °C. The final concentration of TCS in the cultures was analysed using HPLC. The degradation ratios (%) were calculated according to the formula (1) described above.

In addition, samples were taken from the cultures in Processes I, II, and III at the end of the specified incubation period and spread onto TSA medium in petri dishes. After an incubation period of 24 h, the colony and cell morphologies of the bacteria developing on TSA were examined. According to the results obtained, the capacity of co-cultured bacteria to dominate the indigenous microorganisms of wastewater was evaluated.

### Statistical analysis

Three independent experiments were performed and the measurements were made in at least two replicates (*n* = 6). Statistical analysis of the data was performed using SPSS 29.0 and GraphPad Prism 8.0 programs. One-way analysis of variance (ANOVA) was applied for comparisons between groups. Data were expressed as mean ± standard deviation (Mean ± SD). A p-value < 0.05 was considered statistically significant.

## Results and discussion

### Isolation and screening of triclosan-degrading bacteria

In this study, two important points were taken into consideration during the isolation of bacteria capable of degrading triclosan (TCS). The first point was to select high medium pH (pH 8), thereby eliminating moulds, yeasts and fungi. The second point was to add TCS into the medium as sole carbon source during all isolation experiments. The aim of the second approach was to canalize bacteria to use TCS as a carbon source and thus enable them to degrade TCS enzymatically. Considering these points, a total of 24 bacterial isolates capable of degrading TCS were obtained from the activated sludge sample of the wastewater treatment plant.

The TCS degradation capacities of the isolates were determined by spectrophotometric method. The results showed that three isolates (AEM2, AEM5 and AEM20) had higher TCS degradation ability compared to the others. The degradation ratios of TCS were determined to be 59.15% and 59.21% in the culture of the isolate AEM2, 61.9% and 59.86% in the culture of the isolate AEM5, and 20.73% and 18.34% in the culture of the isolate AEM20 according to the spectrophotometric and HPLC-based methods, respectively. These results indicate that spectrophotometric method-based data were in parallel with HPLC-based data. It is well known that spectrophotometric method is easier and requires less labor than the HPLC measurement method. Accordingly, the amount of residual TCS in the cultures was determined spectrophotometrically during optimization studies.

**Fig. 1:**
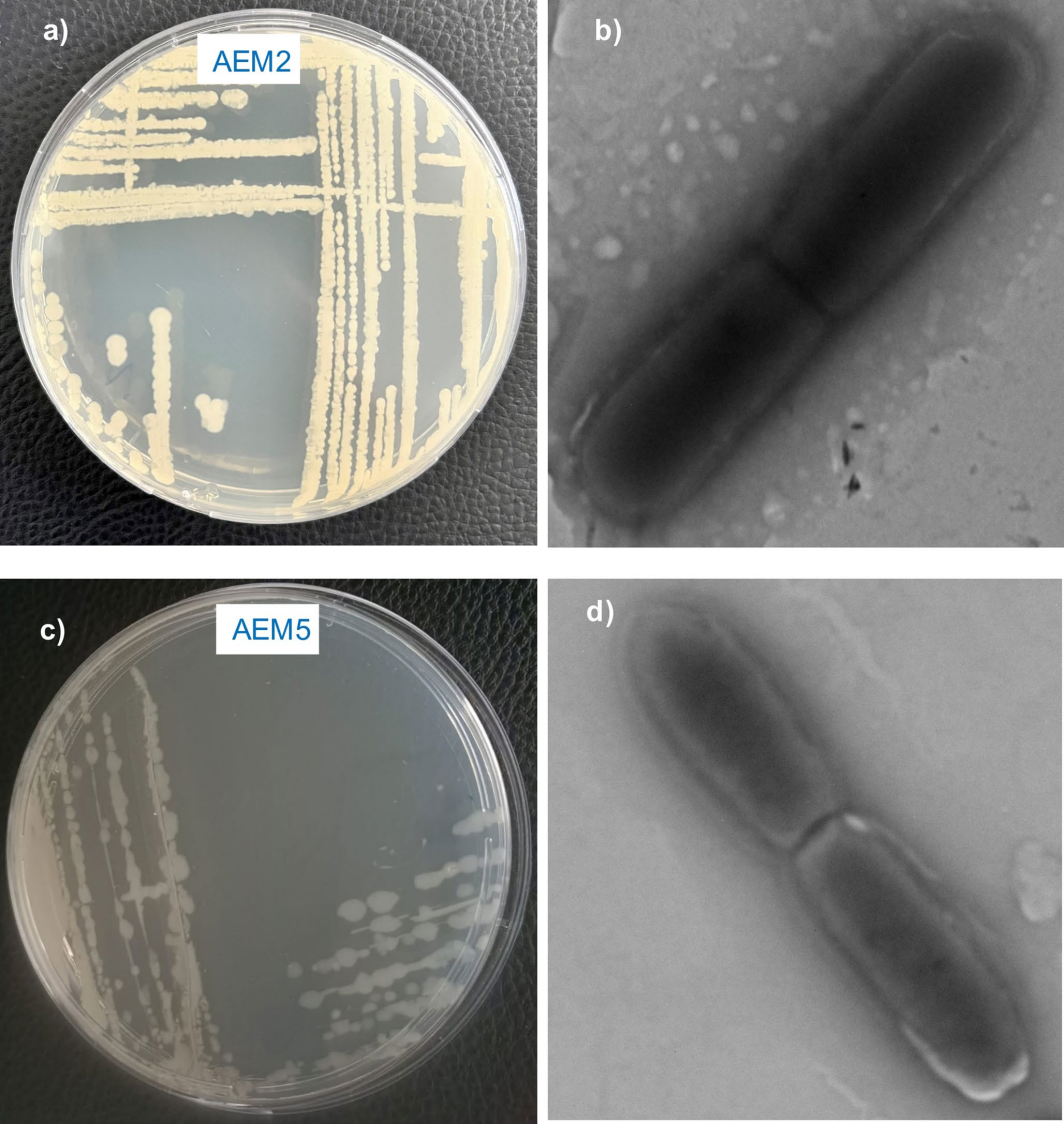
Colony and cell morphologies of AEM2 and AEM5 **a** colony morphology of AEM2 on LB agar, **b** cell morphology of AEM2 based on transmission electron microscope (TEM), **c** colony morphology of AEM5 on LB agar, **d** cell morphology of AEM5 based on TEM

The results also clarified that two isolates (AEM2 and AEM5) have much higher biodegradation ability compared to the others isolates including AEM20 (Supplementary Table S1 and Supplementary Fig. S1). Considering these results, the isolates AEM2 (Fig. 1a) and AEM5 (Fig. 1c) were chosen for the subsequent stages of the study. Both AEM2 and AEM5 were determined to be gram-positive. Transmission electron microscope (TEM) analyses revealed that both isolates are rod-shaped bacteria (Fig. 1b, d).

### Molecular identification of the isolates AEM2 and AEM5

**Fig. 2:**
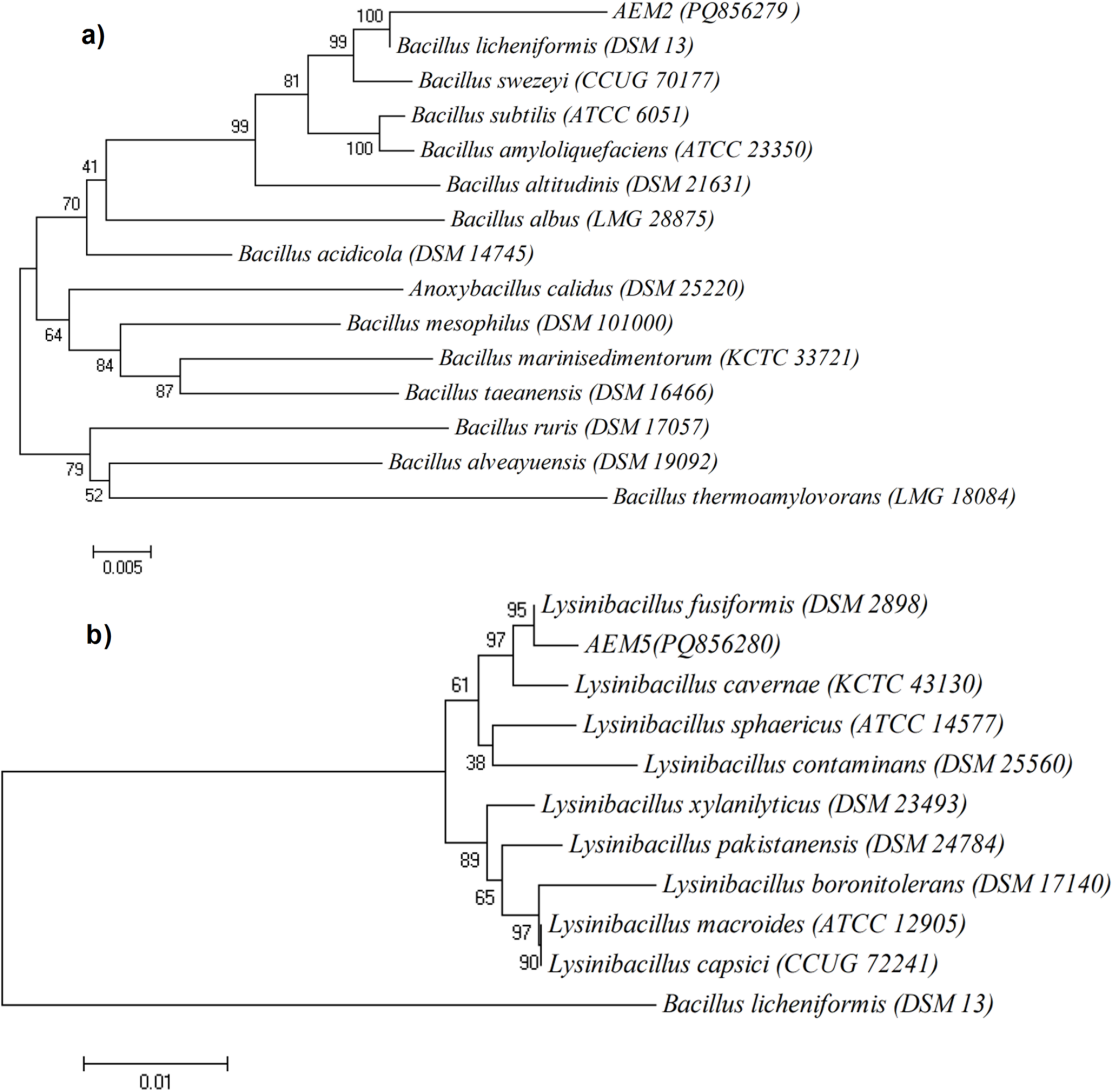
Neighbor joining phylogenetic trees of AEM2 (**a**) and AEM5 (**b**) on the basis of 16 S rRNA gene sequence analysis

Based on 16 S rRNA gene analysis, AEM2 (GenBank accession: PQ856279) and AEM5 (GenBank accession: PQ856280) were identified as *Bacillus licheniformis* (Fig. 2a) and *Lysinibacillus fusiformis*, respectively (Fig. 2b). This finding is good agrement with the fact that *B. licheniformis* and *L. fusiformis* strains can be isolated from wastewater systems [46, 47].

In the literature, it is known that *B. licheniformis* strains are used in the production of various metabolites that find applications in the food, aquaculture, biomedicine, and pharmaceutical industries. Moreover, its some strains are also used in agricultural studies and enviromental biotechnology [48, 49]. Similarly, *L. fusiformis* strains have been reported to find applications in agricultural and industrial biotechnologies as well as environmental biotechnology [50–52].

In environmental biotechnology, strains of two bacteria can be used for removal of heavy metals and the biodegradation of toxic organic compounds [53–57]. On the contrary, there is no report on the TCS degradation potential of neither *B. licheniformis* nor *L. fusiformis*. Therefore, revealing the TCS degradation abilities of these two species may contribute to environmental biotechnology studies.

### Biodegradation of TCS by mono- and co-cultures of selected bacteria

The mono-cultures or co-cultures of microorganisms can be used in biodegradation of organic pollutants; however, co-cultures have been reported to exhibit higher biodegradation capacity [24, 25]. For instance, Li et al. [58] reported that the co-culture containing LM1 and LY1 bacterial strains could degrade approximately 98% of 17β-estradiol (5 mg/L) within 7 days, while the mono-cultures of LM1 and LY1 exhibited the degradation yields of 77% and 68%, respectively. Hong et al. [59] showed that the co-culture of two bacteria (*Rhodococcus rhodochrous* BX2 and *Pseudomonas* sp. LY-1) provided higher triclocarban degradation when compared to their mono-cultures. Wu et al. [59] found that the mono-cultures of *Bacillus subtilis* and *Pseudomonas aeruginosa* could degrade respectively 32.61% and 54.35% of crude oil, while the co-culture of the bacteria exhibited the degradation efficiency of 63.05% However, a co-culture formulation of microorganisms for biodegradation of TCS has not been tested in any study so far. Accordingly, in the present study, the potential of co-culture of two isolates (*B. licheniformis* AEM2) and (*L. fusiformis* AEM5) in TCS degradation was also assessed. The efficiency of the co-culture was compared with mono-cultures of the isolates. During the experiments, the total inoculum volume of both mono-cultures and co-cultures was adjusted to 1 mL/100 mL.

**Fig. 3:**
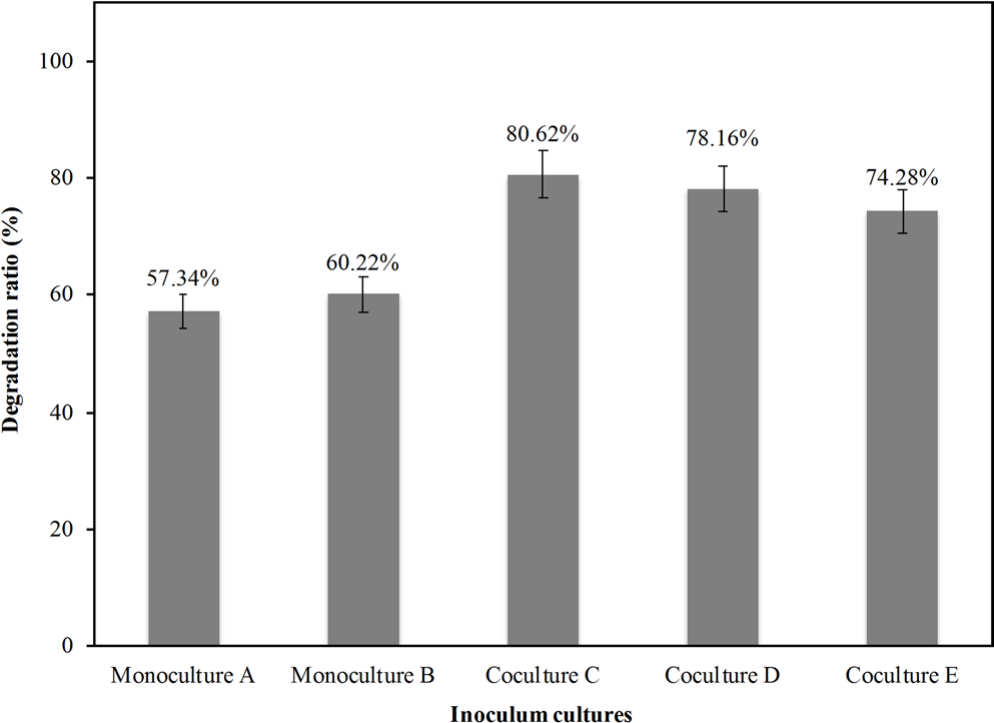
Triclosan-degrading capacities of mono-cultures and co-cultures of isolates. Culture conditions: pH 8, temperature 25 °C, TCS concentration 10 mg/L and incubation time 24 h. The pre-cultures were mono-culture A (1 mL of AEM2 pre-culture), mono-culture B (1 mL of AEM5 pre-culture), co-culture C (0.25 mL of AEM2 pre-culture + 0.75 mL of AEM5 pre-culture), co-culture D (0.5 mL of AEM2 pre-culture + 0.5 mL of AEM5 pre-culture) and co-culture E (0.75 mL of AEM2 pre-culture + 0.25 mL of AEM5 pre-culture). Three independent experiments were conducted and measurements were made in at least two replicates (*n* = 6) and presented as mean ± SD. The residual TCS in the culture was analyzed spectrophotometrically. HPLC analysis was performed only for the cultures of the three isolates that showed TCS degradation ability in spectrophotometric measurements. All statistical analyses were performed using concentration data (mg/L). However, degradation efficiency was presented as percentage removal for comparison clarity

The spectrophotometric results elucidated that mono-culture A (AEM2) and mono-culture B (AEM5) could degrade TCS by approximately 57% (57.34) and 60% (60.22%), respectively. The degradation efficiencies of co-culture C (0.25 mL of AEM2 pre-culture + 0.75 mL of AEM5 pre-culture), co-culture D (0.5 mL of AEM2 pre-culture + 0.5 mL of AEM5 pre-culture), and co-culture E (0.75 mL of AEM2 pre-culture + 0.25 mL of AEM5 pre-culture) were determined as 80.62%, 78.16% and 74.28% at the end of 24 h incubation period, respectively (Fig. 3). These results demonstrate that all co-cultures, especially co-culture C, provided statistically higher biodegradation rates as compared to mono-cultures (*p* < 0.05). As reported in previous studies, the higher degrading capacity of co-culture in the present study can be explained by synergistic effects between two bacteria, namely, mechanisms such as increased enzyme diversity, intermediate product consumption, and stress load sharing [24, 25, 60–63]. Taking into account of these data, the next steps of the work were continued with the co-culture C.

### Effects of environmental factors on TCS biodegradation potential of co-culture

In studies on the degradation of toxic substances by microorganisms, the pH of the medium is one of the important parameters that are effective for the growth and degradation of microorganisms. For example, the study performed by Wang et al. [19] manifested that *Dyella* sp. WW1 isolated from activated sludge exhibited the maximum degradation efficiency at 15 °C and pH 7. Balakrishnan and Mohan [64] reported that the TCS degradation potential of the strain *Providencia rettgeri* MB-IIT was at maximum at pH 7. Kumari et al. [21] elucidated that the TCS-degrading potency of *Citrobacter freundii* KS2003 isolated from a wastewater sample reached to the maximum at pH 8 In the study by Li et al. [65], *Bacillus* sp. DL4 was reported to exhibit the maximum TCS removal at pH 7.31. Temperature is another environmental factor affecting biodegradation efficiency of microorganisms. For example; Ghafouri et al. [23] reported the optimum temperature for TCS removal with *Enterobacter cloacae* as 32 °C. In a separate study [65], the optimum temperature for TCS removal with *Bacillus* sp. DL4 was found to be 35 °C. In the study conducted by Kumari and co-workers [21], the optimum temperature for TCS-degrading ability of *Citrobacter freundii* KS2003 was determined to be 30 °C.

In the present study, the potential of the co-culture to degrade TCS was studied at different (pH 6–10), temperatures (15–60 °C), TCS concentrations (5–50 mg/L) and incubation times (0–96 h). Degradation efficiency was presented as percentage removal for comparison clarity. However, all statistical analyses were performed using concentration data (mg/L).

**Fig. 4:**
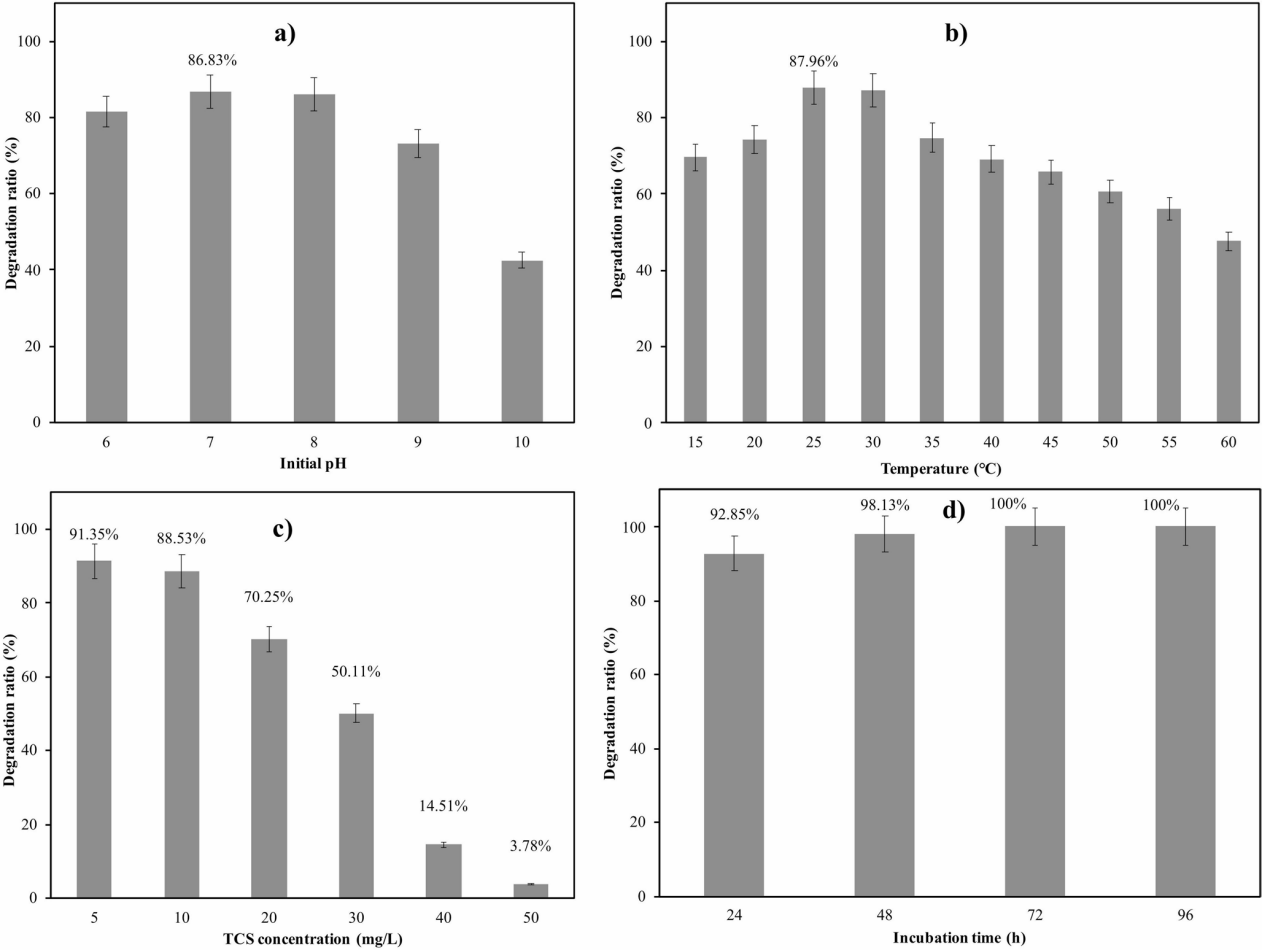
Effects of initial pH (**a**), temperature (**b**), TCS concentration (**c**) and incubation time (**d**) on TCS-degrading potential of co-culture. Effect of initial pH was studied at a temperature of 25 °C, a TCS concentration of 10 mg/L, and an incubation time of 24 h. Effect of temperature was studied at pH 7, TCS concentration of 10 mg/L and an incubation time of 24 h. Effect of TCS concentration was studied at pH 7, a temperature of 25 °C and an incubation time of 24 h. Effect of incubation time was studied at pH 7, a temperature of 25 °C and a TCS concentration of 10 mg/L. Three independent experiments were conducted and measurements were be made in at least two replicates (*n* = 6) and presented as mean ± SD. TCS degradation was analyzed spectrophotometrically. All statistical analyses were performed using concentration data (mg/L). However, degradation efficiency was presented as percentage removal for comparison clarity

Quantitative statistical analysis demonstrated that TCS degradation efficiency of the co-culture was significantly affected by the alterations in the studied operational parameters (*p* < 0.05). TCS degradation by the co-culture was detected across all tested pH conditions, with the highest degradation efficiency (86.83%) observed at pH 7. When the experiments were performed at a TCS concentration of 10 mg/L, an incubation period of 24 h, and pH 7, the co-culture displayed its maximum TCS degradation efficiency (87.96%) at 25 °C, followed by 30 °C. However, it was determined that the co-culture was also capable of degrading TCS in a wide temperature range from 15 to 60 °C (Fig. 4b). Overall, these findings suggest that the co-culture may be used as a novel bioremediation formulation in biological wastewater treatment systems, particularly in environments characterized by variable pH and temperature regimes.

As seen from Fig. 4c, the degradation efficiency decreasd as initial TCS concentration increased (*p* < 0.05), when other operational parameters were kept constant (initial pH 7, temperature 25 °C, and incubation time 24 h). The co-culture was able to degrade 4.57 mg (91.35%) of 5 mg/L TCS. However, a degradation efficiency dropped to 8.853 mg/L (88.53%) at a concentration of 10 mg/L TCS (Fig. 4c). This finding indicates a possible toxic effect of TCS on bacterial growth at higher concentrations. However, the subsequent stages of the study were conducted at the TCS concentration of 10 mg/L, which is the most commonly used concentration in the literature and also is the accumulation rate of TCS in nature.

Another parameter affecting the efficiency of microbial degradation is the incubation time. In the present study, the degration ratios of TCS (10 mg/L) were found to be 92.85%, 98.13%, 100% and 100% at 24, 48, 72 and 96 h, respectively (Fig. 4d). Statistical analyses based on the quantitative data of these degration ratios revealed that incubation time had a significant effect on TCS degradation (*p* < 0.05). The degration ratios and quantitative data elucidated that the co-culture exhibited the highest TCS degradation capacity within the first 24 h of incubation and was able to completely degrade TCS within 72 h.

Studies to date have shown that, depending on the type of bacteria used, the degradation time of TCS ranges from 4 to 13 days [20, 21, 23, 65–67]. Conversely, the results of the current elucidated that the co-culture completely degraded TCS within a shorter time of 72 h. This can be atributed to the high enzymatic activity and rapid metabolic capacity of two bacteria, which are components of the co-culture. The co-culture’s ability to rapidly degrade TCS can shorten processing times in wastewater treatment systems, leading to savings in energy and operational costs.

### Analysis of TCS degradation byproducts and TCS degrading-enzyme activities

After the complete degradation (100%) of TCS was achieved within 72 h, the culture was centrifuged and the supernatant was used for GC-MS based metabolite analysis. The GC-MS analysis revealed that there were 25 substances in the final culture. The concentration of residual TCS (the compound 11 in GC-MS chromatogram) was determined to be only 1.76%, indicating that TCS was almost completely degraded in the culture (Supplementary Fig. S2).

In GC-MS chromatogram, there are some metabolites (Hexadecanoic acid, 9-Octadecanamide, Octadecanoic acid, Nonadecanol, 1-Heptadecane and 1-octadecene), which are considered to be related with cell or lipid metabolism. The compound pyrrolo[1,2-a]pyrazine-1,4-dione, hexahydro-3-(phenylmethyl) possessing a peptide-like structure was considered to be a microbial metabolite. Furthermore, GC-MS chromatogram indicates that the culture contains also small phenolic compounds, with or without Cl.

Microbial enzymes such as laccases, manganese peroxidases, oxygenases and dehalogenases are known to be effective in biodegradation of various toxic compounds including pharmaceuticals, personal care products, phenols, pesticides, herbicides, industrial dyes, etc [39, 64, 68–71]. Laccases and manganese peroxidases are known for their strong degradation activity, high oxidative power, and broad substrate specificity. Therefore, these enzymes or microorganisms-producing them are widely used in bioremediation studies [39]. Oxygenases are another prominent group of microorganisms-derived enzymes related with the biodegradation of toxical aromatic compounds. They catalyze the ring cleavage of the aromatic compounds and thus cause their precise mineralization. There are two main groups of oxygenases: monooxygenases and dioxygenases. The first group catalyzes the incorporation of one atom of the oxygen into the substrate, while the second group perform the catalyzing the addition of both atoms of the oxygen into the substrate [72]. Monooxygenases can perform, C-C bond forming and C-C bond breaking, demethylation and hydroxylation reactions, while dioxygenases catalyze the dehydroxylation reactions [39]. Due to these potential activities, oxygenases (monooxygenases and dioxygenases) and laccases can perform ether bond cleavage in organic pollutants [31, 39, 73].

Dehalogenation is the removal of halogen atoms such as F, Cl, and Br from an organic molecule [71, 74]. In aerobic-biological dehalogenation reactions, hydrolytic dehalogenases and oxygenases are involved. Hydrolytic dehalogenases generally break the C–Cl bond via hydrolysis, namely, they provide hydrolytic dechlorination, and thus play an important role in environmental degradation and biotechnological applications [75]. Some oxygenases can destabilize halogenated substrates via hydroxylation reaction, thus leading to the removal of the halogen from the structure of destabilized subtrates [74].

It has been documented that *B. licheniformis* and *L. fusiformis* are capable of producing hydrolytic enzymes such as manganese peroxidases, laccases, dehalogenases, and oxygenases, which are responsible for toxic organic pollutants [22, 76–80]. The results of the current study revealed that neither laccase nor manganase peroxidase activity was detected in the culture during the incubation period. This finding suggests that neither of these two enzymes plays a role in the breakdown of TCS. Therefore, enzymes being responsible for TCS degradation were predicted based on the chemical reactions catalyzing the formation of TCS degradation by-products.

In the present study, the chlorinated intermediates and characteristic isotope distribution patterns observed in the GC-MS chromatogram indicate that an oxygenase-mediated activation initially occurred on the aromatic ring. Monooxygenase or dioxygenase type enzymes cause oxidative activation by adding a hydroxyl group to the aromatic ring. This event facilitates the cleavage of the ether bond (Ar–O–Ar) [81–83]. In the present study, this oxidative activation carried out by monooxygenase/dioxygenase enzymes may have facilitated the cleavage of the diphenyl ether bond and paved the way for the formation of chlorinated phenolic intermediates. The mono- and di-chlorinated phenols observed in the GC-MS at the mid-retention time suggest that the ether bond of TCS was enzymatically cleaved, thus converting TCS into two separate phenolic derivatives. Similar chlorinated intermediates derived from TCS have also been reported in studies conducted in previous years [84–86].

The detection of mono- and dichlorinated phenols in the GC-MS chromatogram and the weakening of the isotopic pattern parallel to the decrease in chloride number support sequential dehalogenation steps. These results are consistent with the knowledge that dehalogenases and cytochrome P450-type monooxygenases play a role in the oxidative conversion and sequential dehalogenation of chlorinated phenols [87–91]. The presence of lower molecular weight phenol-like compounds during the intermediate retention time indicates that aromatic ring modification or partial ring simplification reactions have occurred.

In short, the proposed pathway for the degradation of TCS involves the following steps: (i) oxygenase-mediated aromatic activation, (ii) oxidative cleavage of the ether bond, and (iii) stepwise dehalogenation leading to the formation of low-chlorinated or chlorine-free phenolic intermediates.

### Cytotoxicity of TCS and its degradation byproducts

The present study revealed that the co-culture of *B. licheniformis* AEM2 and *L. fusiformis* AEM5 effectively degraded TCS. However, ensuring the environmental safety of biodegradation process requires that the resulting byproducts possess lower or no toxicity compared to the parent compound [39, 92, 93]. Therefore, the toxicity of TCS and its degradation products were investigated in this study.

**Fig. 5:**
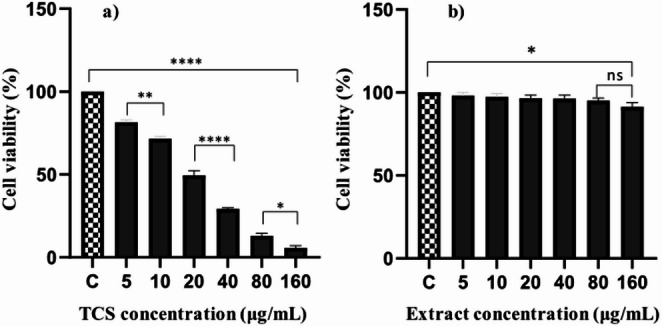
Toxicity analysis of TCS (**a**) and TCS degradation products (**b**) on fibroblasts for 24 h. Cells not exposed to TCS or its degradation products were considered as the control (C) group. Means of three independent experiments with triplicate replicates were calculated in GraphPad Prism 8.0 and presented as mean ± SD. (*) symbol indicates statistically significant changes. *p < 0.05 (significant); **p < 0.01 (highly significant); ***p < 0.001 and ****p < 0.0001 (highly significant)

The ethyl acetate extract from the culture was determined to contain 160 µg of substance. It was thought that this extract was mainly composed of the breakdown products of TCS, since only mineral salts were initially added to the medium. The extract was dissolved in 1 mL DMSO and this final material was used as a stock solution to analyze the breakdown products of TCS. Similarly, a stock solution of 160 µg TCS was prepared in 1 mL DMSO. Then, both stock solutions were diluted with 1% DMSO to prepare the dilutions at different concentrations (5–160 µg/mL DMSO). The toxicity of the prepared dilution samples was tested on L929 cells (mouse fibroblast cell line) by WST-1 cell viability assay. The cytotoxicity assesment demonstrated that in comparison to the control (no treatment), TCS treatment statistically decreased cell viability at all concentrations tested (*p* < 0.05). The cell viability was measured as 5% at the highest concentration (160 µg/mL) of TCS, while 81% cell viability was detected at the lowest concentration (5 µg/mL), indicating a concentration-dependent cytotoxic effect of TCS. In contrast to TCS, its degradation products did not cause a statistically significant decrease in cell viability within the concentration range of 5–80 µg/mL (*p* > 0.05). A marginal cytotoxic effect of degradation products was detected only at 160 µg/mL (Fig. 5).

In the literature, it has been documented that bacterial consortia can metabolize pollutants such as pesticides, personal care products and textile dyes, thereby causing a concomitant decrease in their cytotoxicity [39, 94, 95]. In consistent with the findings of previous studies, the findings of this study indicate that the co-culture metabolizes TCS into the metabolites with substantially lower cytotoxic potential. This situation, as indicated in previous studies, can be explained by the decrease in the Cl content of TCS and its conversion into smaller phenolic metabolites [11, 18, 96]. Collectively, these comparative data suggest that from a toxicological standpoint, the co-culture system of *B. licheniformis* AEM2 and *L. fusiformis* AEM5 appears to be a promising and environmentally safe candidate for bioremediation applications.

### Biodegradation of TCS by co-coculture in non-sterile wastewater-based medium

Microbial biodegradation of organic pollutants can be achieved in non-sterile-culture processes [37, 40–44]. In a non-sterile culture process, wastewater medium is not sterilized and external contamination is not controlled during the biodegradation. Therefore, this process diminishes labor and energy consumption [33, 39]. However, there is no report on the bacterial degradation of TCS in a non-sterile process. Moreover, it is stated that in comparison to mono-cultures of microorganisms, their respective co-cultures show higher biodegradation ability under non-sterile conditions [24, 28–31, 37]. Accordingly, in the last step of this study, the TCS biodegradation potency of the co-culture was examined under non-sterile culture conditions that simulate the conditions of real biological treatment systems.

For this purpose, three different processes were designed. In all processes, the medium consisted of only wastewater and TCS (10 mg/L), without the addition of external carbon, nitrogen, mineral, or vitamin sources. This approach was selected to evaluate the feasibility of the designed process under nutrient-limited conditions, thereby reducing operational costs related with nutrient supplementation.

The first difference between the three processes was whether the medium was sterilized or not, and the second difference was whether co-culture was inoculated into the medium. In the process I, the medium was sterilized to kill endogenous microorganisms and then was inoculated with co-culture. In this process, it was aimed to evaluate the ability of the co-culture to degrade TCS under sterile conditions. In the process II, the medium was not sterilized and directly inoculated with the co-culture. The aim of using this process was to evaluate the TCS-degrading capacity of the co-culture under non-sterile conditions in the presence of endogenous microorganisms. In fact, the main process intended to be designed in the present study was process II. In the process III, the medium was not sterilized and the co-culture was not inoculated. The purpose of using process III was to evaluate the potency of the endogenous microorganisms of wastewater to degrade TCS. The HPLC measurements showed that TCS degradation was 98.77% in the process I, 88.55% in the process II, and 1.1% in the process III (Table 1 and Supplementary Fig. 3S). A low degradation ratio of 1.1% in the process III may be explained by the presence of indigenous microorganisms in the wastewater. These results indicate a slight decrease in degradation efficiency under non-sterile conditions (Process II) compared to sterile conditions (Process I). However, the high removal rate of Process II (88.55%) demonstrates that the co-culture successfully competes with the indigenous microorganisms and maintains considerable biological degradation activity. This may be attributed to competition between the co-cultured microorganisms and the indigenous microorganisms of the wastewater environment in terms of factors such as dominance and access to the substrate.

**Table 1:** TCS-degrading potential of co-culture under sterile and non-sterile conditions

Process	External nutrients	Medium sterilization	Co-culture inoculation	Contamination control	Degradation ratio (%)
Process I	–	+	+	+	98.77
Process II	–	–	+	–	88.55
Process III	–	–	–	–	1.1

In all processes, the medium consisted only wastewater and 10 mg/L TCS. The initial pH of the media was not adjusted (the initial pH of the wastewater was 7.12). The flasks were incubated in a shaking incubator at 150 rpm for 72 h. The concentration of residual TCS in flasks was determined using HPLC. All statistical analyses were performed using concentration data (mg/L). However, degradation efficiency was presented as percentage removal for comparison clarity

The fact that Process II (88.55%) provided higher degradation efficacy than Process III (1.1%) indicates that the co-cultured bacteria suppressed indigenous microorganisms and dominated the wastewater environment. To support this finding, samples were taken from the cultures in Processes I, II, and III at the end of 72 h incubation period and spread onto TSA medium in petri dishes. After incubating petri dishes for 24 h, bacteria developing on TSA were examined for their colony and cell morphologies. The results revealed that the culture medium of process I contained only the colonies of the co-cultured *B. licheniformis* AEM2 and *L. fusiformis* AEM5. This situation may be attributed to the fact that the wastewater medium was subjected to a sterilization process before being inoculated with a co-culture of the two bacteria. It was determined that co-cultured *B. licheniformis* AEM2 and *L. fusiformis* AEM5 accounted for the majority of bacteria in the culture II (Process II), and there are only a few bacterial colonies originating from wastewater. The culture III (process III) contained only wastewater-derived bacteria, which have different cell and olony morphologies. These results suggest that the co-culture-based process II was able to suppress indigenous microorganisms in wastewater (Supplementary Fig. S4).

It is known that wastewater sterilization is not practical on an industrial scale, and its supplementation with external nutrients increases sludge production and downstream processing costs [39, 97]. The results from process II indicate that the co-culture was able to degrade TCS in wastewater medium without such interventions. Therefore, this finding may be significant from a scale-up and industrial application perspective. However, other operational parameters such as continuous-flow conditions, oxygen transfer efficiency, long-term microbial community dynamics and transformation products-mediated toxicity analysis should be also tested in future studies to validate the effectiveness of the co-culture-based process II. These interventions will contribute to the translation of laboratory findings into practical applications and the implementation of the designed co-culture system in real wastewater treatment systems.

## Conclusions

In the present study, two triclosan-degrading bacterial strains belonging to *Bacillus licheniformis* and *Lysinibacillus fusiformis* were isolated from activated sludge samples taken from a wastewater treatment plant. The co-culture of two isolates were determined to have higher TCS-degrading capacity than their respective mono-cultures under the tested culture conditions. In vitro preliminary toxicity tests indicated that the degradation products of TCS did not cause toxic effects on skin fibroblasts within the tested concentration range. Additionally, the co-culture showed TCS-degrading potential in a wastewater-based medium designed to simulate real conditions of biological treatment systems. Preliminary degradation tests also suggested that the co-culture may perform TCS degradation in a wastewater-based medium without pH, temperature, sterilization control, and nutrient supplementation. However, further studies are necessary to confirm its operational performance, cost-effectiveness and environmental safety under real wastewater treatment conditions. Overall, this study provide a basis for future studies exploring the development of microbial co-culture-based strategies for the removal of TCS.

## Supplementary Information

Below is the link to the electronic supplementary material.

Supplementary Material 1

## Data Availability

The data used to support the findings of this study are included within the manuscript and supplementary material.
