# Supplementary material for: Biodegradation of triclosan under non-sterile conditions by co-culture of Bacillus licheniformis and Lysinibacillus fusiformis
Source: Bioprocess Biosyst Eng. 2026 Apr 18;49(6):1497–512. doi: 10.1007/s00449-026-03331-9 (PMC13328321; doi:10.1007/s00449-026-03331-9)
Supplement: Supplementary file 1 — Supplementary Material 1 [file 449_2026_3331_MOESM1_ESM.docx]

**Biodegradation of triclosan under non-sterile conditions by co-culture of *Bacillus licheniformis* and *Lysinibacillus fusiformis***

**Short title:** Biodegradation of triclosan by a bacterial co-culture

**Table S1. Screening of TCS-degrading bacteria**

| Isolate code | Spectrophotometric assay | HPLC assay |
| --- | --- | --- |
| AEM1 | 17.34% | Not Analysed |
| AEM2 | 59.15% | 59.21% |
| AEM3 | 6.61% | Not Analysed |
| AEM4 | 14.51% | Not Analysed |
| AEM5 | 61.90% | 59.86% |
| AEM6 | 18.47% | Not Analysed |
| AEM7 | 12.82% | Not Analysed |
| AEM8 | 11.69% | Not Analysed |
| AEM9 | 6.04% | Not Analysed |
| AEM10 | 0.96% | Not Analysed |
| AEM11 | 14.51% | Not Analysed |
| AEM12 | 10.56% | Not Analysed |
| AEM13 | 10.56% | Not Analysed |
| AEM14 | 19.60% | Not Analysed |
| AEM15 | 8.870% | Not Analysed |
| AEM16 | 15.64% | Not Analysed |
| AEM17 | 2.09% | Not Analysed |
| AEM18 | 19.03% | Not Analysed |
| AEM19 | 17.34% | Not Analysed |
| AEM20 | 20.73% | 18.34% |
| AEM21 | 4.91% | Not Analysed |
| AEM22 | 7.74% | Not Analysed |
| AEM23 | 16.21% | Not Analysed |
| AEM24 | 12.25% | Not Analysed |

*Culture Conditions: pH 8, temperature 25°C, TCS concentration 10 mg/L and incubation time 24 h. Three parallel experiments were performed in three different flasks for each isolate. Spectrophotometric measurements were performed twice for each flask (n=6). HPLC analysis was performed only for the cultures of three isolates that showed TCS degradation ability in spectrophotometric analyses.

**HPLC results of TCS-degrading potentials of AEM2, AEM5 and AEM20 isolates**

According to the results obtained from HPLC, the percentage of TCS biodegradation was calculated. For this purpose, the following formula was used (1).

$Degradation ratio \left( \% \right)=\left( \frac{C0-C1}{C0} \right)X 100 (1)$ Where C_0_ is the initial TCS concentration (mg/L) and C_1_ is the final TCS concentration (mg/L).

**Figure S1a.** HPLC results of TCS-degrading potential of the isolate AEM2

**Figure S1b.** HPLC results of TCS-degrading potential of the isolate AEM5

**Figure S1c.** HPLC results of TCS-degrading potential of the isolate AEM20

**Figure S2.** GC-MS result of TCS and its degradation byproducts

**Biodegradation of TCS by co-culture under sterile and non-sterile conditions**

In the study, three different processes were designed. In all processes, the medium was contained only wastewater and TCS (10 mg/L). In the process I, the medium was sterilized to eliminate endogenous microorganisms and then was inoculated with co-culture. In the process II, the medium was not sterilized and directly inoculated with coculture. In the process III, the medium was not sterilized and the co-culture was not inoculated. The final concentration of TCS in the cultures was analysed using HPLC. The degradation ratios (%) were calculated according to the formula (1) described above.

**Figure S3a. Biodegradation of TCS using process I**

**Figure S3b. Biodegradation of TCS using process II**

**Figure S3c. Biodegradation of TCS using process III**

**
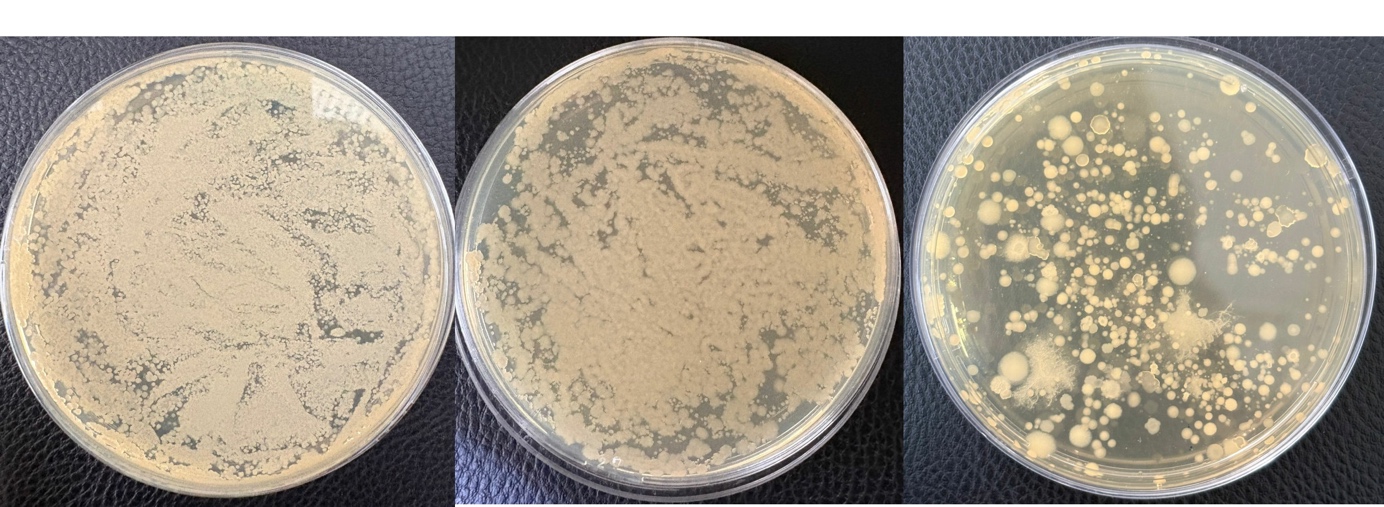
**

**Figure S4. Bacterial diversity in cultures from Processes I-III.** Samples were taken from the cultures in Processes I, II, and III at the end of 72 h incubation period and spread onto TSA medium in petri dishes. After incubating petri dishes for 24 h, bacteria developing on TSA were examined for their colony and cell morphologies. The results revealed that the culture medium of process I contained only the colonies of the co-cultured *B. licheniformis* AEM2 and *L. fusiformis* AEM5. It was determined that co-cultured *B. licheniformis* AEM2 and *L. fusiformis* AEM5 accounted for the majority of bacteria in the culture II (Process II), and there are only a few bacterial colonies originating from wastewater. The culture III (process III) contained only wastewater-derived bacteria, which have different cell and olony morphologies.
